# Impact psychologique de la pandémie COVID-19 sur les patients hémodialysés chroniques dans l’oriental marocain: étude transversal

**DOI:** 10.11604/pamj.2024.48.129.44064

**Published:** 2024-07-24

**Authors:** Kaouthar El Mir, Salah-Eddine El Jabiry, Meryem Errabehy, Yassamine Bentata, Fatima Elghazouani, Bouchra Oneib

**Affiliations:** 1 Service de Psychiatrie, Hôpital de la Santé Mentale et des Maladies Psychiatriques, Centre Hospitalo-Universitaire Mohammed VI, Oujda, Maroc,; 2 Faculté de Médecine et de Pharmacie Oujda, Université Mohammed Premier, Oujda, Maroc,; 3 Laboratoire de Recherche sur la Santé Materno-Infantile et Mentale, Faculté de Médecine et Pharmacie Oujda, Université Mohammed Premier, Oujda, Maroc,; 4 Service de Néphrologie, Centre Hospitalo-Universitaire Mohammed VI, Oujda, Maroc,; 5 Laboratoire d'Epidémiologie, Recherche Clinique et Santé Publique, Faculté de Médecine et de Pharmacie Oujda, Université Mohammed Premier, Oujda, Maroc

**Keywords:** COVID-19, chronic haemodialysis patients, mental disorder, quality of life, Morocco, COVID-19, chronic haemodialysis patients, mental disorder, quality of life, Morocco

## Abstract

**Introduction:**

les hémodialysés chroniques sont une population fragile, qui ont été extrêmement influencée par la pandémie COVID-19 qui a eu des répercussions graves chez les personnes ayant des comorbidités et une dépression de système immunitaire ce qui expose davantage les hémodialysés chroniques à un haut risque infectieux et de développer une forme grave de COVID-19, ce présent travail vise à évaluer l'impact de la pandémie COVID-19 sur la santé mentale et sur la qualité de vie des hémodialysés chroniques.

**Méthodes:**

enquête transversale descriptive, réalisée auprès de 175 hémodialysés chroniques. Nous avons utilisé l'échelle Mini International Neuropsychiatric Interview Moroccan Arab Version 5.0.0, échelle de stress perçu et échelle de la qualité de vie des hémodialysés chroniques (KDQOL-SFTM 1.3).

**Résultats:**

cent soixante-quinze (175) participants ont été recrutés, dont 76 patients ont été atteints du COVID-19. L'infection au COVID-19 était significativement liée à l'âge (p=0,018) aux troubles psychiatriques (p=0,00), à un antécédent de tentative de suicide (p=0,006) et au niveau élevé du stress (p=0, 01). La qualité de vie des hémodialysés chroniques était significativement altérée chez les malades atteints du COVID-19 (p=0,00), surtout chez les sujets âgés (p=0,034), vivant seuls (p=0,004), ayant des antécédents organiques (p=0,04), psychiatriques (p=0,00), ou toxiques (p=0,003), ainsi que chez les malades présentant une forme symptomatique (p=0,001), des complications (p=0,00), ou hospitalisation secondaire à COVID-19 (p=0,00), et chez ceux ayant un stress sévère (p=0,00).

**Conclusion:**

la santé mentale et la qualité de vie des hémodialysés chroniques ont été essentiellement influencées négativement au cours de la pandémie COVID-19.

## Abstract (en)

**Introduction:**

chronic haemodialysis patients are a vulnerable population significantly affected by the COVID-19 pandemic, which has had a severe impact on people with co-morbidities and immune system depression, which increases the risk of infection and of developing severe form of COVID-19. The purpose of this study is to assess the impact of the COVID-19 pandemic on the mental health and quality of life of chronic haemodialysis patients.

**Methods:**

we conducted a descriptive cross-sectional survey among 175 chronic hemodialysis patients using the Mini International Neuropsychiatric Interview Moroccan Arab Version 5.0.0 scale, the Perceived Stress Scale, and the Quality of Life Scale for Chronic Hemodialysis Patients (KDQOL-SFTM 1.3).

**Results:**

one hundred and seventy-five (175) participants were recruited, of whom 76 patients had COVID-19. COVID-19 infection was significantly associated with age (p=0.018), psychiatric disorders (p=0.00), a history of suicide attempts (p=0.006) and high-stress levels (p=0.01). The quality of life of chronic haemodialysis patients was significantly impaired in patients with COVID-19 (p=0.00), especially in subjects who were elderly (p=0.034), lived alone (p=0.004), had a history of organic (p=0.04), psychiatric (p=0.00), or substance abuse issues (p=0.003), as well as in patients with a symptomatic form (p=0.001), complications (p=0.00), or hospitalisation secondary to COVID-19 (p=0.00), and those with severe stress (p=0.00).

**Conclusion:**

the mental health and quality of life of chronic haemodialysis patients were mainly negatively influenced during the COVID-19 pandemic.

## Introduction

Une crise sanitaire grave en lien avec une nouvelle maladie virale responsable d'une épidémie d'un syndrome respiratoire aigu sévère-corona virus-2 (SRAS-CoV-2) (COVID-19) qui est un nouveau brin de la famille des coronavirus [1]. Cette épidémie qui a frappé tout le monde, est une menace humaine majeure qui s'est transformée en pandémie [2], Notre pays, comme d'autres, vit également cette crise depuis début mars 2020 [3]. Le 11 mars 2020, la situation a été déclarée comme une pandémie et état d'urgence sanitaire par l'OMS [4]. Ce nouveau coronavirus présente une morbidité particulièrement élevée chez les personnes âgées et les populations présentant des comorbidités qui peuvent avoir des répercussions graves [5], ce qui a intensifié les craintes à l'échelle mondiale dans la prestation des soins à cause de son taux d'infection extrêmement élevé et sa mortalité relativement importante surtout au sein de la population immunodéprimée, entre autres chez les hémodialysés chroniques qui sont à haut risque infectieux [6], et de développer en conséquence une forme grave de la maladie ce qui a créé un défi devant tout patient hémodialysé chronique atteint du COVID-19 chez les soignants néphrologues dont la prise en charge thérapeutique devient lourde et le pronostic vital de ces malades peut se mettre en jeu [7].

Au cours de cette pandémie, des mesures de confinement ont été retenus afin de limiter la propagation de ce virus et contrôler la surmortalité, néanmoins, les hémodialysés chroniques ne pouvant être confinés, dont chacun a besoin de 2 ou 3 séances par semaines mais cela était difficile à respecter parce qu'ils envisageaient des contraintes pour se déplacer afin de continuer leur suivi médical et garder la régularité de leurs séances d'hémodialyse. Les hémodialysés chroniques souffrent d'une altération psychique par divers symptômes d'anxiété et de dépression [8], Et cela est secondaire à plusieurs facteurs fréquemment retrouvées comme comorbidités chez les patients hémodialysés tels que les pathologies sous-jacentes, la fréquence des hospitalisations, la douleur chronique, les troubles du sommeil, la fatigue accrue et la dépendance aux soins notamment en milieu hospitalier, ainsi que le risque de développer une forme grave et fatale d'infection par COVID-19 ce qui aggrave davantage leur état psychique [9]. C'est un sujet auquel beaucoup de chercheurs se sont intéressés, ce présent article cherche à démontrer l'impact de la pandémie COVID-19 sur la qualité de vie des hémodialysés chroniques et de décrire comment elle peut affecter la santé mentale et physique tout en analysant les facteurs qui contribuent à ces effets.

L'objectif de ce travail est d'étudier les scores de la qualité de vie et de l'échelle de Mini ainsi que l'échelle de stress perçu chez les patients hémodialysés chroniques en période de pandémie COVID-19 afin de souligner l'impact psychologique de la pandémie sur cette population vulnérable. Hypothèse nulle (H0): La pandémie de COVID-19 n'a pas d'impact significatif sur la santé mentale et sur la qualité de vie des hémodialysés chroniques. Hypothèse alternative (H1): la pandémie de COVID-19 a un impact négatif significatif sur la santé mentale et sur la qualité de vie des hémodialysés chroniques.

## Méthodes

**Cadre de l'étude:** il s'agit d'une étude transversale descriptive, qui vise à évaluer l'impact de la pandémie COVID-19 sur la qualité de vie et sur la santé mentale des hémodialysés chroniques.

**Contexte organisationnel et participants:** cette étude a été réalisée auprès des patients hémodialysés chroniques et effectuée au niveau du centre d'hémodialyse Hassan II de la région de l'oriental marocain, étalée sur une période de 2 ans à partir de la fin de 2021 à 2023.

**Critères d'inclusion:** les deux sexes sont concernés, présence d'un consentement éclairé, patients faisant de l'hémodialyse chronique durant la pandémie COVID-19, un âge de plus de 18 ans et patients ayant les capacités mentales pour participer à l'étude en répondant de façon consciente et claire aux items du questionnaire.

**Critères d'exclusion:** âge est inférieur à 18 ans, patients récemment inclus dans l'hémodialyse et patients suivis pour des troubles psychiatriques avant la pandémie COVID-19.

**Variables:** les variables étudiées dans notre présente étude se composent principalement d'une variable dépendante, qui est l'infection au COVID-19, et de plusieurs variables indépendantes, qui sont les suivantes: l'âge, le sexe, la situation matrimoniale, les antécédents personnels médicochirurgicaux, les antécédents personnels et familiaux psychiatriques, toxiques et de tentative de suicide.

### Source de données et de mesures

***Sources de données:*** les données ont été fusionnées dans une base de données à l'aide du logiciel SPSS Inc © Statistical Package for the Social Sciences (version 21) et stockées sur un ordinateur protégé par un mot de passe.

***Sources de mesures:*** nous avons choisi trois échelles:

**Echelle de MINI:** *International Neuropsychiatric Interview (M.I.N.I.)* est un entretien diagnostique, valide et fiable. Nous avons travaillé avec la version du *M.I.N.I* traduite et validée en dialectale arabe marocain dont sa validité et sa fiabilité ont été testée à l'aide d'un avis d'un expert en psychiatrie dont les résultats de l'étude de validité et de fiabilité étaient très satisfaisants [10]. Elle avait pour but dans notre étude l'évaluation psychique des hémodialysés chroniques et le dépistage des troubles psychiatriques.

**Echelle de stress perçu:** cette échelle est l'une des plus utilisées pour évaluer la perception subjective du stress. Elle comporte 10 items qui permettent d'évaluer l'importance avec laquelle des situations de la vie sont perçues comme menaçantes. Sa validité est confirmée par nombreuses études qui ont démontré sa robustesse et sa sensibilité pour détecter les différences individuelles dans la perception du stress [11]. Son interprétation subdivise les degrés de stress en 3 classes: *stress gérable:* score inférieur à 21; *stress modéré:* score compris entre 21 et 26 et *stress sévère:* score supérieur à 27.

**Echelle de la qualité de vie KDQOL-SFTM 1.3:** c'est un instrument d'auto-évaluation des personnes atteintes de maladies rénales et en dialyse, jugé valide et fiable par de nombreuses études [12,13]. Elle permet d'évaluer la composante physique, psychique, sociale et rénale, il comprend 43 items ciblés sur la maladie rénale ainsi que 36 items qui fournissent l'évaluation de l'état de santé général (RAND 36-Item Heath Survey 1.0 ou SF-36TM). Nous avons utilisé la version valide au Maroc de l'échelle de KDQOL-SF TM 1.3 traduite en dialecte marocain [14]. Son interprétions est quantitative et se fait comme suit: *scores élevés:* indiquent que la qualité de vie est meilleure et *scores faibles:* indiquent que la qualité de vie est altérée.

**Biais:** garantie de l'anonymat des participants pour réduire les biais de déclaration. Nous avons utilisé des échelles validées pour une exploration globale de la composante psychique et physique à l'aide de l'échelle de stress perçu ainsi que l'échelle de la qualité de vie chez les hémodialysés chroniques (KDQL-SFTM version 1,3) et l'échelle de MINI, ses dernières ont été abordées en dialecte marocain avec des versions validées au Maroc pour réduire le biais d'information. Mise en œuvre de techniques statistiques appropriées pour contrôler les biais potentiels dans l'analyse des données.

**Taille de l'échantillon:** nous avons réalisé un recrutement exhaustif de tous les enquêtés.

**Analyse statistique:** l'analyse statistique a été effectuée avec le logiciel SPSS Inc © *Statistical Package for the Social Sciences (version 21)* par le service d'épidémiologie et de recherche clinique de la faculté de médecine. L'analyse statistique s'est déroulée en deux étapes descriptives et comparatives.

**Une analyse descriptive:** les variables qualitatives sont présentées en termes de pourcentages (les tranches d'âge, le sexe, le statut marital, le mode vie, les antécédents organiques et psychiatriques, les antécédents de tentative de suicide, consommation des substances psychoactives, le degré du stress). Les variables quantitatives en termes de moyenne et Ecart-type (Age et la qualité de vie).

**Une analyse comparative:** pour étudier l'impact de la pandémie COVID-19 et ses facteurs associés, nous avons divisé notre échantillon en deux groupes:

***Groupe 1:*** hémodialysés chroniques atteints de l'infection COVID-19 (n=76 cas) (43,4%).

***Groupe 2:*** hémodialysés chroniques non atteints de l'infection COVID-19 (n= 99 cas) (56,6%)

La comparaison des pourcentages a été faite par le test de Chi2 ou le test exact de Fisher et nous avons utilisé le test de *Student* pour la comparaison des 2 moyennes et la méthodes ANOVA pour la comparaison de plusieurs moyennes. Le seuil de signification est fixé à 0,05. La normalité et la linéarité ont été vérifiées.

**Considérations éthiques:** le recueil des données a été effectué avec respect de l'anonymat des patients et de la confidentialité de leurs informations tout au long des différents temps de l'étude. Accord favorable du comité d'éthique pour la recherche biomédicale d'Oujda (CERBO): sous la référence: 43/2021.

## Résultats

**Figure 1:**
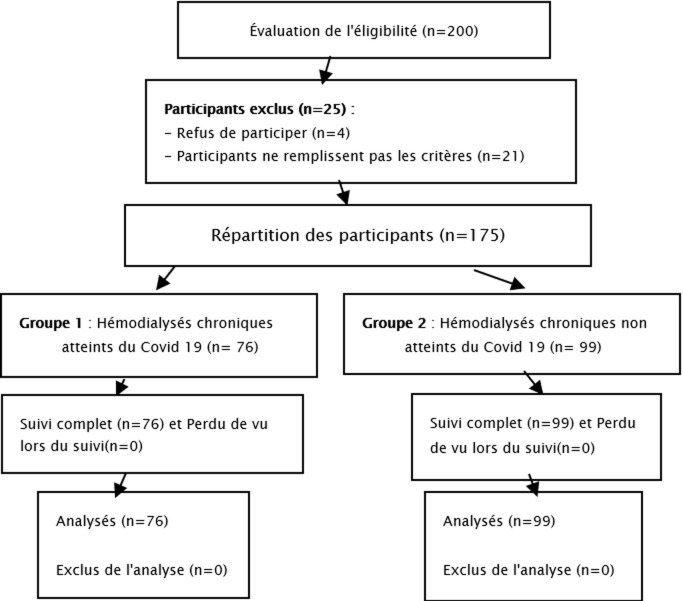
diagramme de flux de la population étudiée

**Participants:** initialement, nous avons eu 200 patients, mais nous n'en avons recruté que 175 patients, en excluant 25 qui ne répondaient pas aux critères d'inclusion dont nous avons envisagé; un refus de participation pour 4 malades et 21 malades ne remplissaient pas les critères dont 5 patients avaient moins de 18 ans, 10 patients étaient récemment inclus à l'hémodialyse et 6 patients étaient déjà suivi pour un trouble psychiatrique avant la pandémie COVID-19 (Figure 1).

**Résultats sociodémographiques:** la moyenne d'âge dans notre échantillon était 53,5 ans ± 15,4 avec des extrêmes allant de 19 à 95 ans (n=175). La majorité des patients de notre échantillon étaient de sexe masculin soit 91 participants (52%), 84 étaient de sexe féminin (48%), avec un sex ratio de 0,92. Dans notre échantillon, 64,5% des participants étaient mariées, 21,14% étaient célibataires, 5,7% étaient divorcées et 8,6% veuves. La majorité des malades réside avec leurs familles (93%), tandis qu'une minorité qui vit seule (7%).

**Résultats descriptifs:** quatre-vingt-quatre virgule six pourcent (84,6%) des hémodialysés chroniques ayant des antécédents de pathologies organiques dont la comorbidité la plus fréquente est l'hypertension artérielle (35,4%), puis vient respectivement le diabète (30,3%), l'hépatite B (12%), la néoplasie (8%), les séquelles d'accident vasculaire cérébral (4%), et l'hépatite C (1,1%). Sur 175 patients recrutés, nous avons noté que 4,6% des hémodialysés chroniques avaient fait au moins une tentative de suicide. Treize virgule sept pourcent (13,7%) des hémodialysés chroniques consomment des substances psychoactives. Quarante-trois virgule quatre pourcent (43,4%) (n=76) des hémodialysés chroniques étaient atteints de l'infection COVID-19, pourtant que 73,7% étaient vaccinés dont la majorité a chopé le virus au cours des séances d'hémodialyse (59,2%). De plus 66% de la population atteinte du COVID-19 présentent une forme symptomatique, 56,6% des patients ont été hospitalisés et 38% des patients avaient des complications organiques suite à l'infection au COVID-19 (Tableau 1).

**Tableau 1:** caractéristiques des patients atteints du COVID-19

Caractéristiques des patients atteints du COVID-19	N = 76 (43,4%)
Vaccination	56 (73,7%)
**Mode de transmission du virus**	
Séance d'hémodialyse	45 (59,2%)
En interfamilial	22 (29%)
Milieu public	7 (9,2%)
Au travail	2 (2,6%)
**Forme symptomatique**	50 (66%)
**Forme asymptomatique**	41 (54%)
**Hospitalisation suite au COVID-19**	43 (56,6%)
**Complication suite au COVID-19**	29 (38%)

Les pathologies psychiatriques selon l'échelle de MINI sont répartis comme suit: une dépression dans 22,3% des cas, un état de stress post traumatique dans 13,7% des cas et un trouble anxieux généralisé dans 5,1% des cas et autres…, Ainsi que 16% des cas ayant un stress sévère selon l'échelle du stress perçu (Tableau 2).

**Tableau 2:** les différents indicateurs mesurés dans la population étudiée

Les indicateurs mesurés	Echantillon étudié (n=175)		Total
	COVID POSITIF N=76 (43,4%)	COVID NEGATIF N= 99 (56,6%)	
**Echelle de MINI**			
Episode dépressif majeur	8 (20,5%)	31(79,5%)	39 (22,3%)
Risque suicidaire	2 (66,7%)	1 (33,3%)	3 (1,7%)
Trouble panique	3 (6%)	2 (40%)	5 (2,8%)
Agoraphobie	1 (100%)	0	1 (0,5%)
Trouble obsessionnel compulsif	7 (100%)	0	7 (4%)
Etat de stress post traumatique post COVID-19	24 (100%)	0	24 (13,7%)
Trouble psychotique	2 (100%)	0	2 (1,1%)
Trouble anxieux généralisé	4 (44,4%)	5 (55,6%)	9 (5,1%)
Trouble bipolaire	1 (100%)	0	1(0,5 %)
**Echelle de stress perçu**			
Gérable	40 (35%)	73 (65%)	113 (64,5%)
Modéré	18 (53%)	16 (47,1%)	34 (19,4%)
Sévère	18 (64%)	10 (36%)	28 (16%)
**Echelle de la qualité de vie des hémodialysés chroniques**			
La moyenne de la qualité de vie	46,5 ± 15	55,3 ±17	47,2 ± 20

La qualité de vie moyenne des hémodialysés chroniques (n=175) était 47,2 ± 20, qui varie entre 22 et 86 dont chez ceux qui ont été atteints du COVID-19, la QDV moyenne était à 46,5 ± 15 et varie entre 22 et 75,5, et celle des malades non atteints du COVID-19 était à 55,3 ± 17 et varie entre 23,55 et 86.

Le score le plus bas a été obtenu pour la composante mentale qui était à 46,68 ± 20,3, et le score moyen le plus élevé a été obtenu pour la composante des symptômes et problèmes en rapport avec la maladie rénale 74 ± 15,77 (Tableau 3).

**Tableau 3:** scores des différentes échelles de la qualité de vie chez toute la population

Echelles	Moyenne	Médiane	Ecart-type	Minimum	Maximum
Score physique	47,77	44,16	21,10	9,38	93,33
Score mental	46,68	44,16	20,30	10,50	84,38
Symptômes et problèmes en rapport avec la maladie rénale	74	77,08	15,77	16,67	100
Poids de la maladie rénale	46,84	45,83	23,11	0	93,75
Conséquence de la maladie rénale	52,85	52,04	12,30	26,42	83,26
Satisfaction de la prise en charge	59,84	66,66	15,61	19,44	88,90
N.B: plus le score est élevé, la qualité de vie est meilleure					

**Analyse univariée:** l'infection au COVID-19 a atteint la population étudiée progressivement avec l'âge (p=0,018), et ceux qui vivent en familles (p=0,46), ainsi que ceux ayant des comorbidités organiques (p=0,7), les consommateurs des substances psychoactives (p=0,48), ceux ayant des troubles psychiatriques (p =0,00), et antécédent de tentative de suicide (p=0,006). Le niveau élevé de stress est fortement lié à l'infection au COVID-19 (p= 0, 01) (Tableau 4). La qualité de vie chez les hémodialysés chroniques atteints du COVID-19 est significativement plus basse (p= 0,00). Elle s'altère significativement avec l'âge (p=0,034), chez les patients qui vivent seuls (p= 0,004), chez ceux qui ont des comorbidités organiques (p=0,04), consommateurs des substances psychoactives (p=0,03), surtout les tabagisme (p=0,04), ainsi que ceux ayant une forme symptomatique du COVID-19 (p= 0,01), et ayant eu des complications organiques suite à l'infection (p=0,00) et avaient une nécessité d'une hospitalisation (p= 0,00).

**Tableau 4:** facteurs associés à l’infection COVID-19

Variables	Echantillon étudié (n= 175) (100%)		Odds Ratio (Intervalle de confiance à 95%)	P
	COVID POSITIF (n=76) (43,4%)	COVID NEGATIF (n=99) (56,6%)		
**Tranche d'âge**				
18-38	40,5%	59,5%		0,018
39-58	39%	61%		
59-78	47%	53%		
79-98	100%	0		
**Sexe**				
Homme	43%	57%	1,05 [0,57;1,9]	0,47
Femme	44%	56%		
**Statut marital**				
Célibataire	37,8%	62,2%		0,32
Marié (e)	42,5%	57,5%		
Divorcé (e)	70%	30%		
Veuf (Ve)	46,7%	53,3%		
**Mode de vie**				
Seul	33%	67%	1,58 [0,45-5,46]	0,46
En famille	44%	56%		
**Comorbidités organiques**				
Oui	44%	56%	1,1[0,4-2,921]	0,70
Non	41%	59%		
**HTA**				
Oui	45%	55%	0,89[0,48-1,067]	0,75
Non	42,5%	57,5%		
**Diabète**				
Oui	47%	53%	1,24[0,65-2,37]	0,51
Non	42%	58,2%		
**Trouble psychiatrique**				
Oui	57%	43 %		0,0000
Non	28,6%	71%	3,1[1,16-0,56]	
**Tentative de suicide**				
Oui	75%	25%	4,15[0,8; 21,2]	0,006
Non	42%	58 %		
**Habitudes addictives**				
Oui	50%	50%	1,35 [0,57;3,22]	0,48
Non	42,5%	57%,5		
**Echelle de stress perçu**				
Gérable	35%	65%		0,01
Modéré	53%	47,1%		
Sévère	64%	36%		
**La moyenne de la qualité de vie**	46,5 ± 15	55,3 ±17		0,00

La moyenne de la qualité de vie chez les hémodialysés chroniques atteints du COVID-19 parait significativement élevée chez ceux n'ayant pas de trouble psychiatrique personnel (p=0,00) ou familial (p= 0,001) ou des idées suicidaires (p= 0,027). Une association significative entre le stress sévère et l'altération de la qualité de vie (p=0,00) (Tableau 5).

**Tableau 5:** facteurs des risques influençant la qualité de vie chez les hémodialysés chroniques atteints du COVID-19

Variables	Qualité de vie moyenne	P
**Âge**		0,034
18-38	53 ± 15,2	
39-58	45,4 ±14,2	
59-78	45 ± 6,2	
79-98	40,4 ± 5,9	
**Sexe**		0,62
Homme	47,3 ± 16	
Femme	45,7 ± 14,5	
**Statut matrimonial**		0,1
Célibataire	42 ± 9,35	
Marié (e)	49 ± 16	
Divorcé (e)	51 ± 17	
Veuf (Ve)	37 ± 8	
**Mode de vie**		0,004
Seul (e)	39 ± 5	
En famille	47 ± 15	
**Comorbidités organiques**		0,04
Oui	42 ± 20,5	
Non	47 ± 14	
**Troubles psychiatriques**		0,00
Oui	40 ± 11,6	
Non	61 ± 11,4	
**Antécédents des idées suicidaires**		0,027
Oui	43 ± 7	
Non	47 ± 15,5	
**Trouble psychiatrique dans la famille**		0,001
Oui	40 ± 10,5	
Non	51 ± 16	
**Consommation des SPA**		0,03
Oui	38 ± 12,6	
Non	48 ± 15	
**Tabagisme**		0,04
Oui	37,5 ± 14	
Non	48 ± 15	
**Forme symptomatique**		0,01
Symptomatique	43 ± 13,2	
Asymptomatique	53 ± 16,3	
**Complications organiques secondaires au COVID-19**		0,00
Oui	36,6± 10	
Non	54 ± 14,3	
**Hospitalisation secondaire à COVID-19**		0,00
Oui	39 ± 9,3	
Non	56,4 ± 15	
**Stress perçu**		0,00
Gérable	54 ± 15	
Sévère	38 ± 9	

## Discussion

La pandémie COVID-19 est une flambée mondiale récente et fatale raison pour laquelle nous avons pensé à évaluer la santé mentale et la qualité de vie qui ont été fortement influencées chez notre population étudiée au cours de cette période. Cependant nous avons confronté des limites méthodologiques dont l'entretien et le recueil des données d'exploitation étaient délicats d'autant plus que les malades se fatiguaient ce qui peut être un biais d'information. De plus, la petite taille de l'échantillon et le fait que l'étude soit monocentrique peuvent introduire un biais de sélection. Ainsi que les formes asymptomatiques des infections au COVID-19 peuvent passer inaperçues chez certains patients, ce qui pourrait entraîner des cas positifs manqués et introduire un biais de surveillance.

Les patients hémodialysés chroniques sont susceptibles d'être infectés par le COVID-19 plus particulièrement chez ceux qui ont un âge avancé et cela a été constaté dans une étude a été menée sur 59 hémodialysés chroniques [15], ainsi que dans notre présente étude dont le risque d'infection au COVID-19 augmente avec l'âge.

Les hémodialysés chroniques ont souvent besoin de soins de la part de membres de leur famille ou de soignants, et si un soignant est infecté, il peut transmettre le virus à tous les contacts étroits, y compris le patient hémodialysé, ce qui augmente le risque de contamination par le virus [16], et cela a été également prouvé dans notre étude dont les hémodialysés chroniques qui vivent en famille sont plus atteints du COVID-19 par rapport à ceux qui vivent seuls ( 44 Vs 33; p= 0,46).

La présence des comorbidités organiques favorise la sévérité d'un épisode infectieux à SARS-CoV-2 [17] et augmente le risque du taux de mortalité [18], ainsi que dans notre présente étude, les hémodialysés chroniques ayant eu des comorbidités organiques étaient plus atteint par COVID-19 (44% VS 41%) surtout les diabétiques (47% VS 42%) et les hypertendus (45% VS 42,5%). À cela s'ajoute que le diabète [19], et l'hypertension [20] et l'atteinte rénale [21], peuvent mettre en jeu le pronostic vital des malades atteints du COVID-19.

Dans l'hémodialyse chronique, comme il y'a des complications somatiques il y'a des perturbations psychiques [22] par conséquent l'hémodialysé chronique peut s'impacter facilement par la pandémie COVID-19 qui avait des répercussions psychiques graves [23,24], dans notre présente étude nous avons constaté une association significative entre les troubles psychiatriques chez les hémodialysés chroniques et l'infection au COVID-19 ( 57% VS 28,6%; p =0,00) dont le trouble de stress post traumatique est le plus retrouvé chez les hémodialysés chroniques atteints du COVID-19. Il est décrit dans la littérature qu'il représente une complication assez fréquente de l'infection de SARS-Cov2 [25] avec une augmentation des troubles dépressifs de 27,6% et une augmentation des troubles anxieux de 25,6% [26] au cours de la pandémie COVID-19, ainsi qu'une forte association entre la séropositivité au coronavirus et la survenue de symptômes psychotiques a aussi été rapportée [27].

De nombreux facteurs peuvent altérer la santé mentale et majorer l'émergence d'idées suicidaires et le passage à l'acte suicidaire au cours de la pandémie COVID-19, dans une étude a été portée sur 5186 participants au cours de cette crise sanitaire, a objectivé que 11,9% ont déclaré avoir sérieusement envisagé de se suicider [28], à cela s'ajoute la distanciation et le confinement [29], ainsi que l'exposition aux agents infectieux a été reconnue comme facteur aggravant le risque de passage à l'acte autoagressif [30] et cela a été également constaté dans notre enquête dont nous avons noté une association significative entre l'infection au COVID-19 et les conduites suicidaires (p= 0,006). Certaines recherches récentes supposent que le virus COVID-19 est fortement impliqué dans le risque de conduite suicidaire dont ce virus se lie à l'enzyme de conversion qui peut être subir à des remaniements génomiques qui sont associés au comportement suicidaire [31,32].

Les fumeurs peuvent être exposés à un risque accru de contracter le virus en raison d'une fonction pulmonaire altérée, d'un système immunitaire affaibli [33] et cela se concorde également avec notre présente étude dont les hémodialysés chroniques consommateurs sont plus exposé à l'infection COVID-19 par rapport à ceux qui ne consomment pas (50% vs 42,5%; p= 0,48).

Les hémodialysés chroniques ont vécu des niveaux de stress beaucoup élevés d'autant plus au cours de cette période critique de COVID-19, qui sont liées soit à l'épidémie, au confinement, à la peur de contracter le virus COVID-19 [34], dans une étude menée auprès des hémodialysés chronique au cours de cette crise sanitaire, les auteurs ont constaté que le score d'anxiété était chez 37,7% [35], en outre le stress et l'altération de la santé mentale favorisent l'affaiblissement de l'immunité et l'exposition aux différentes pathologies à savoir [36]; auto immunes [37], infectieuses, néoplasiques [38], et cela a été également constaté par notre présente enquête dont nous avons objectivé que les hémodialysés chroniques ayant un niveau de stress sévère sont plus exposé au risque infectieux COVID-19 vis-à-vis ceux qui ont un stress gérable (64% Vs 36%; p=0,001). En outre un stress psychologique prolongé et des situations stressantes telles que celles rencontrées dans la pandémie COVID-19 augmentent l'abus de substances ce qui affecte la qualité de vie en générale ainsi que les situations stressantes peuvent diminuer les réponses immunitaires aux infections, ainsi que la réponse à la vaccination en général [39].

La pandémie COVID-19 était un facteur bouleversant de la qualité de vie des hémodialysés chroniques dont dans notre présente étude nous avons objectivé une diminution de la qualité de vie chez les hémodialysés chronique qui était à 47,25 ± 19,87 par rapport à une étude qui a été menée en Tunisie en 2016 [40] et réalisée avant la pandémie COVID-19 en 2016, dont la qualité de vie des hémodialysés était à 51,6 ± 14. En outre, dans notre enquête nous avons constaté une baisse remarquable des scores des différents données de la qualité de vie des hémodialysés chroniques par rapport à des résultats qui ont été obtenus avant la pandémie COVID-19 dans une étude qui a traité la qualité de vie chez les hémodialysés chroniques aux états unis (USA) [41], donc la qualité de vie des hémodialysés chroniques au cours de la pandémie COVID-19 a été essentiellement influencée négativement, ainsi que par l'âge avancé au-delà de 60 ans [40] et cela a été également constaté dans notre récente étude dont la qualité de vie des hémodialysés chroniques atteints du COVID-19, s'altère significativement avec l'âge (p=0,034) et ceci peut être expliqué non seulement par la détérioration de la santé physique mais aussi par le fléchissement des capacités adaptatives générales chez le sujet âgé [42]. En outre la vie en famille représente aux hémodialysés un facteur positif qui contribue au bien être amélioré [43-45], et cela a été aussi constaté dans notre étude nous avons objectivé que les hémodialysés qui vivent en famille ayant une meilleure qualité de vie (47±15 Vs 39±5; p=0,04).

C'est compréhensible que l'augmentation du nombre des comorbidités affecte négativement la qualité de vie chez les hémodialysés chroniques [46], et ceci se concorde complétement avec ce que nous avons trouvé dans notre travail dont la présence des comorbidités quoique ce soit psychiques (p=0,00) ou somatiques (p=0,00), ou forme symptomatique de l'infection COVID-19 (p=0,01) avec des complications de COVID-19 (p=0,00) et la nécessité d'une hospitalisation secondaire au COVID-19 (p=0,00), altèrent et diminue significativement la qualité de vie. Le tabagisme est un facteur de risque reconnu de survenue d'un grand nombre de pathologies: cardiovasculaires, bronchopneumopathie, infectieuses [47], pour ces nombreuses raisons dans notre présente étude, la qualité de vie chez les hémodialysés chroniques atteints du COVID-19 et consommateurs du tabac avaient une baisse significative de la qualité de vie (p= 0,04). En outre, l'altération de la santé mentale influence la qualité de vie des hémodialysés chroniques [48,49], et cela a été également affirmé par notre récente étude dont nous avons également constaté une baisse significative de la qualité de vie chez les malades ayant un stress sévère par rapport à ceux qui ont un stress gérable (38 ± 9 Vs 54± 15; p= 0,00).

Les résultats obtenus dans notre étude peuvent ne pas être généralisables à d'autres régions ou pays avec des conditions socio-économiques et sanitaires différentes. Cependant, cette crise sanitaire a touché le monde entier, y compris les patients hémodialysés chroniques dont leur prise en charge est similaire à l'échelle mondiale. Ces patients ont vécu des situations stressantes similaires pendant la pandémie de COVID-19, telles que le risque d'irrégularité des séances d'hémodialyse en raison du confinement et la gravité accrue du risque infectieux en raison de leurs nombreuses comorbidités somatiques et psychiques. Tous ces facteurs nous permettent de bien connaitre le degré d'impact de cette pandémie sur la santé mentale et la qualité de vie des hémodialysés chroniques.

## Conclusion

La santé mentale et la qualité de vie des hémodialysés chroniques au cours de cette pandémie ont été essentiellement influencées négativement, ainsi que par d'autres facteurs associés tel que l'âge avancé, les comorbidités, la consommation des substances psychoactives, et le niveau élevé de stress. Il nous semble donc indispensable de bien réfléchir et agir à cette problématique en mettant en place des stratégies de prévention et un accompagnement psychologique à cette catégorie de malades fragiles.

### Etat des connaissances sur le sujet

- *La pandémie COVID-19 est une flambée mondiale récente et fatale qui a répercuté beaucoup de dégâts sur tous les plans (social, économique, médical …);*
- *L'impact de la pandémie COVID-19 sur l'organisation générale et sur les soins au niveau des centres d'hémodialyse;*
- *Les patients hémodialysés chroniques sont exposés à haut risque infectieux et ayant plusieurs comorbidités.*

### Contribution de notre étude à la connaissance

- *L'infection au COVID-19 a un fort impact sur la santé mentale des hémodialysés chroniques;*
- *La qualité de vie des hémodialysés chroniques a été négativement influencée par la pandémie COVID-19;*
- *Peu de recherches scientifiques ont traité l'impact psychologique secondaire à l'infection COVID-19 chez cette catégorie de malades.*

## Remerciements

Les auteurs tiennent à remercier tout le personnel du centre d'hémodialyse Hassan II de la région de l'oriental marocain. Ils remercient également les malades, qui malgré le fardeau de leur maladie ont accepté de participer à cette étude.

## References

[1] Dhama K, Khan S, Tiwari R, Sircar S, Bhat S, Malik YS (2020). Coronavirus Disease 2019-COVID-19. Clin Microbiol Rev.

[2] Chan JF-W, Yuan S, Kok K-H, To KK-W, Chu H, Yang J (2020). A familial cluster of pneumonia associated with the 2019 novel coronavirus indicating person-to-person transmission: a study of a family cluster. Lancet.

[3] Ministère de la santé et de la protection sociale Maroc Le Maroc annonce l'enregistrement du premier cas du nouveau Coronavirus (communiqués).

[4] Organization mondiale de la santé (OMS) COVID-19 – Chronologie de l'action de l'OMS.

[5] Hacker KA, Briss PA, Richardson L, Wright J, Petersen R (2021). COVID-19 and Chronic Disease: The Impact Now and in the Future. Prev Chronic Dis.

[6] Beaudreuil S, Hebibi H, Charpentier B, Durrbachr A (2008). Les infections graves chez les patients en dialyse péritonéale et en hémodialyse chronique conventionnelle : péritonites et infections de la voie d´abord vasculaire. Réanimation.

[7] Su K, Ma Y, Wang Y, Song Y, Lv X, Wei Z (2020). How we mitigated and contained the COVID-19 outbreak in a hemodialysis center: Lessons and experience. Infect Control Hosp Epidemiol.

[8] Wang L-J, Che C-K, Polenakovic M The Psychological Impact of Hemodialysis on Patients with Chronic Renal Failure. Renal Failure - The Facts 2012 InTech.

[9] Mengin A, Allé MC, Rolling J, Ligier F, Schroder C, Lalanne L (2020). Conséquences psychopathologiques du confinement. Encephale.

[10] Kadri N, Agoub M, Gnaoui S, Alami K, Hergueta T, Moussaoui D (2015). Mini International Neuropsychiatric Interview Moroccan (Arabic) current DSM IV.

[11] Giorgio MT Echelle de mesure du stress perçu : Perceived Stress Scale, PSS.

[12] Kutner NG (1994). Assessing end-stage renal disease patients' functioning and well-being: measurement approaches and implications for clinical practice. Am J Kidney Dis.

[13] Valderrábano F, Jofre R, López-Gómez JM (2001). Quality of life in end-stage renal disease patients. Am J Kidney Dis.

[14] Joshi VD, Mooppil N, Lim JF (2010). Validation of the Kidney Disease Quality of Life-Short Form: a cross-sectional study of a dialysis-targeted health measure in Singapore. BMC Nephrol.

[15] Valeri AM, Robbins-Juarez SY, Stevens JS, Ahn W, Rao MK, Radhakrishnan J (2020). Presentation and Outcomes of Patients with ESKD and COVID-19. J Am Soc Nephrol.

[16] Basile C, Combe C, Pizzarelli F, Covic A, Davenport A, Kanbay M (2020). Recommendations for the prevention, mitigation and containment of the emerging SARS-CoV-2 (COVID-19) pandemic in haemodialysis centres. Nephrol Dial Transplant.

[17] Khedhiri A, Ben Amar A, Kammoun F (2021). Impact psychologique de la pandémie COVID-19 sur une population d´hémodialysés chroniques en Tunisie : étude monocentrique. Nephrol Ther.

[18] Diawara A, Aminou M, Idrissa E, Adamou F, Adehossi E, Anya B (2022). Comorbidité COVID-19 et maladies chroniques à l´Hôpital général de référence (HGR) de Niamey au Niger. Rev Epidemiol Sante Publique.

[19] Williamson EJ, Walker AJ, Bhaskaran K, Bacon S, Bates C, Morton CE (2020). Factors associated with COVID-19-related death using OpenSAFELY. Nature.

[20] Zheng Y-Y, Ma Y-T, Zhang J-Y, Xie X (2020). COVID-19 and the cardiovascular system. Nat Rev Cardiol.

[21] Cheng Y, Luo R, Wang K, Zhang M, Wang Z, Dong L (2020). Kidney disease is associated with in-hospital death of patients with COVID-19. Kidney Int.

[22] Bossola M, Ciciarelli C, Di Stasio E, Conte GL, Vulpio C, Luciani G (2010). Correlates of symptoms of depression and anxiety in chronic hemodialysis patients. Gen Hosp Psychiatry.

[23] Mallet J, Massini C, Dubreucq J, Padovani R, Fond G, Guessoum SB (2022). Santé mentale et Covid : toutes et tous concernés. Une revue narrative. Ann Med Psychol (Paris).

[24] Losada-Baltar A, Jiménez-Gonzalo L, Gallego-Alberto L, Pedroso-Chaparro MDS, Fernandes-Pires J, Márquez-González M (2021). "We Are Staying at Home" Association of Self-perceptions of Aging, Personal and Family Resources, and Loneliness With Psychological Distress During the Lock-Down Period of COVID-19. J Gerontol B Psychol Sci Soc Sci.

[25] Cherif H, Kalboussi S, Fenina W, Triki M, Yangui F, Charfi MR (2022). État de stress post-traumatique après une infection à COVID-19. Rev Malad Respir Actual.

[26] Xiong J, Lipsitz O, Nasri F, Lui LMW, Gill H, Phan L (2020). Impact of COVID-19 pandemic on mental health in the general population: A systematic review. J Affect Disord.

[27] Severance EG, Dickerson FB, Viscidi RP, Bossis I, Stallings CR, Origoni AE (2011). Coronavirus Immunoreactivity in Individuals With a Recent Onset of Psychotic Symptoms. Schizophr Bull.

[28] Czeisler MÉ, Lane RI, Wiley JF, Czeisler CA, Howard ME, Rajaratnam SMW (2021). Follow-up Survey of US Adult Reports of Mental Health, Substance Use, and Suicidal Ideation During the COVID-19 Pandemic, September 2020. JAMA Netw Open.

[29] Prezelin-Reydit M, Idier L, Combe C, De-Precigout V, Vendrely B, Vigneau C (2020). Impact psychologique du confinement pendant l´épidémie de COVID-19 chez les patients hémodialysés. Nephrol Ther.

[30] Gjervig Hansen H, Köhler-Forsberg O, Petersen L, Nordentoft M, Postolache TT, Erlangsen A (2019). Infections, Anti-infective Agents, and Risk of Deliberate Self-harm and Suicide in a Young Cohort: A Nationwide Study. Biol Psychiatry.

[31] Liji T Microbial characteristics of COVID-19.

[32] Sparks DL, Hunsaker III JC, Amouyel P, Malafosse A, Bellivier F, Leboyer M (2009). Angiotensin I-converting enzyme I/D polymorphism and suicidal behaviors. Am J Med Genet B Neuropsychiatr Genet.

[33] Karila L, Benyamina A (2021). Addictions en temps de pandémie. La Presse Médicale Formation.

[34] Guerraoui A, Idier L, Hallonet P, Dolley-Hitze T, Gosselin M, Duneau G (2021). Répercussions psychologiques du confinement et de l´épidémie à COVID-19 chez les patients et soignants en hémodialyse en France. Nephrol Ther.

[35] Oujidi H, Hamdaoui M, Elalj W, Berkchi F, Bentata Y, Haddiya I (2022). Évaluation du vécu psychologique des patients en hémodialyses chroniques au cours de la pandémie COVID-19. Néphrologie & Thérapeutique.

[36] Jacque C, Thurin J-M (2002). Stress, immunité et physiologie du système nerveux. Med Sci (Paris).

[37] Delévaux I, Chamoux A, Aumaître O (2013). Stress et auto-immunité. Rev Med Interne.

[38] Sklar LS, Anisman H (1981). Stress and cancer. Psychol Bull.

[39] Minihan E, Gavin B, Kelly BD, McNicholas F (2020). COVID-19, mental health and psychological first aid. Ir J Psychol Med.

[40] Zouari L, Omri S, Turki S, Maâlej M, Charfi N, Ben Thabet J (2016). Quality of life in chronic hemodialysis patients: about 71 cases. Tunis Med.

[41] Fukuhara S, Lopes AA, Bragg-Gresham JL, Kurokawa K, Mapes DL, Akizawa T (2003). Health-related quality of life among dialysis patients on three continents: the Dialysis Outcomes and Practice Patterns Study. Kidney Int.

[42] Taji L, Thomas D, Oliver MJ, Ip J, Tang Y, Yeung A (2021). COVID-19 chez les patients ontariens sous dialyse à long terme. CMAJ.

[43] Mingardi G (1998). From the development to the clinical application of a questionnaire on the quality of lifein dialysis, The experience of the Italian Collaborative DIA-QOL (Dialysis-Quality of Life). Nephrol Dial Transplant.

[44] Evans RW, Manninen DL, Garrison LP, Hart LG, Blagg CR, Gutman RA (1985). The quality of life of patients with end-stage renal disease. N Engl J Med.

[45] Nasr M, Hadj Ammar M, Khammouma S, Ben Dhia N, Ghachem A (2008). L'hémodialyse et son impact sur la qualité de vie. Nephrol Ther.

[46] Callaban MB, Funk-Shrag W, Hall L, Mapes DL, Mckevitt P, Molzabn A (2012). Measuring dialysis patients' health-related quality of life with the KDQOL-36Tm. Medical Education Institute.

[47] Le Faou AL, Scemama O (2005). Épidémiologie du tabagisme. Rev Mal Respir.

[48] Vázquez I, Valderrábano F, Fort I, Jofré R, López-Gómez JM, Moreno F (2004). [Differences in health-related quality of life between male and female hemodialysis patients]. Nefrologia.

[49] Njah M, Nasr M, Ben Dhia N (2001). Morbidite anxio-dépressive chez le patient hémodialysé. Nephrologie.

